# Evaluation of the Feasibility of Endothelial Colony-Forming Cells to Develop Tissue-Engineered Vascular Grafts Based on the Gene Expression Profile Analysis

**DOI:** 10.17691/stm2022.14.3.02

**Published:** 2022-05-28

**Authors:** E.A. Velikanova, M.Yu. Sinitsky, А.V. Sinitskaya, V.G. Matveeva, М.Yu. Khanova, L.V. Antonova

**Affiliations:** Researcher, Laboratory for Cell Technologies; Research Institute for Complex Issues of Cardiovascular Diseases, 6 Sosnovy Blvd, Kemerovo, 650002, Russia; Senior Researcher, Laboratory of Genome Medicine; Research Institute for Complex Issues of Cardiovascular Diseases, 6 Sosnovy Blvd, Kemerovo, 650002, Russia; Researcher, Laboratory of Genome Medicine; Research Institute for Complex Issues of Cardiovascular Diseases, 6 Sosnovy Blvd, Kemerovo, 650002, Russia; Senior Researcher, Laboratory for Cell Technologies; Research Institute for Complex Issues of Cardiovascular Diseases, 6 Sosnovy Blvd, Kemerovo, 650002, Russia; Junior Researcher, Laboratory for Cell Technologies; Research Institute for Complex Issues of Cardiovascular Diseases, 6 Sosnovy Blvd, Kemerovo, 650002, Russia; Head of Laboratory for Cell Technologies; Research Institute for Complex Issues of Cardiovascular Diseases, 6 Sosnovy Blvd, Kemerovo, 650002, Russia

**Keywords:** endothelial colony-forming cells, mononuclear fraction of peripheral blood, coronary artery endothelial cells, gene expression, tissue engineering

## Abstract

**The aim of the study** was to assess the suitability of endothelial colony-forming cells in the development of tissue engineering constructs based on the study of the gene expression profile compared to mature endothelial cells.

**Materials and Methods:**

In the experiment, we used the endothelial colony-forming cells (ECFC) obtained from the peripheral blood of patients who underwent percutaneous coronary intervention. The cells were isolated on a Histopaque 1077 density gradient (Sigma-Aldrich, USA), and then cultured in EGM-2MV culture medium (Lonza, Switzerland). A commercial culture of primary human coronary artery endothelial cells (HCAEC) was used as a control. The cells were unfrozen and cultured according to the manufacturer’s recommendations in MesoEndo Cell Growth Medium (Cell Applications, USA).

The experiment was carried out in specialized μ-Luer plates in the perfusion system (IBIDI, Germany), which provided a continuous unidirectional flow of the culture medium with a shear stress of 5 dyn/cm^2^. Control plates were cultured under standard conditions for a similar period of time. Total RNA was isolated from cell samples. The expression of the genes *NOTCH4*, *NRP2*, *PLAT*, *PLAU*, *NOTCH1*, *FLT1*, *COL4A2*, *CD34*, *SERPINE1*, *HEY2*, *MKI67*, *KLF4*, *LYVE1*, *FLT4* was assessed using a quantitative real-time polymerase chain reaction. The expression of the genes was calculated by the ΔCt method and expressed on a logarithmic (log10) scale as a fold change relating to the control samples.

**Results:**

In mature endothelial cells HCAEC when exposed to a laminar flow, only the transcription factor *KLF4* and venous differentiation *NRP2* marker values increased significantly. ECFC showed statistically significant growth in *KLF4*, *NRP2*, *CD34*, and *LYVE1*, as well as *PLAU* expression decrease. In addition, we observed the overexpression of *FLT4*, *LYVE1*, *NOTCH4*, and *NRP2* in ECFC in relation to HCAEC and *HEY2* hypoexpression. *CD34* overexpression characteristic of progenitor cells was also found. An increase in *COL4A2* expression associated with type IV collagen synthesis was a characteristic feature of ECFC.

**Conclusion:**

The gene expression profile of endothelial colony-forming cells is quite close to that of primary endothelial cells of the human coronary artery, and thus, the cells obtained from patients’ peripheral blood can be used to develop personalized tissue-engineered constructs.

## Introduction

*In vitro* vascular graft formation is one of the promising approaches in tissue engineering, which is currently a surging trend [1]. The approach suggests cell mass culturing on a scaffold surface with construct formation, which can be implanted into the circulatory bed in order to replace an affected vascular part. Pivotal questions in developing the technique are the selection of optimal cell source, the selection of materials and methods to make a scaffold, the determination of culture best fitted the physiological conditions.

Endothelial colony-forming cells (ECFC) [2] are considered to be a possible attractive source of endothelial cells to be used in tissue engineering since the cells have high angiogenic potential [3, 4]. According to numerous studies, ECFC can be isolated from a great number of sources including blood [5, 6], bone marrow [7], adipose tissue [8], embryonic stem cells [9], and many others. In the terms of further use in clinical practice, the feasibility to differentiate ECFC from mononuclear fraction of peripheral blood attracts much attention. The advantage of such cells compared to mature ones is their high proliferative potential [10], which enables to obtain a great deal of cell mass that is important to form tissue-engineered constructs. Moreover, possible heterogeneity of ECFC from different sources, as well as the effect of a tissue-engineered carrier on their functional properties, call for further studies.

For successful endothelialization of tissue-engineered vascular grafts, it is necessary to create maximally physiological conditions including a shear stress effect on cells. Endothelial cells exposed to a fluid flow is the necessary condition for their normal activity and endothelial monolayer formation [11]. Under laboratory conditions, the pulsative flow simulation is attained by using pumps initiating a culture medium flow with designed parameters including shear stress force.

**The aim of the study** was to investigate ECFC gene expression profile compared to mature endothelial cells to assess the suitability of their usage to develop tissue-engineered vascular grafts.

## Materials and Methods

### Cell culturing

The study was carried out in accordance with Helsinki Declaration (2013) and approved by the Ethics Committee of Research Institute for Complex Issues of Cardiovascular Diseases (Kemerovo, Russia). The patients gave their consent to be involved in the research.

For the experiment, we used ECFC isolated from peripheral blood of patients with coronary heart disease (n=8), who underwent percutaneous coronary intervention. ECFC isolation and fortification were performed according to a modified protocol by Kolbe et al. [12]. A mononuclear fraction was isolated on Histopaque 1077 density gradient (Sigma-Aldrich, USA), then resuspended in EGM-2MV culture medium (Lonza, Switzerland) containing 5% fetal bovine serum (HyClone, USA), and inoculated in collagen-covered culture flasks. After a one-week culture, the cells were taken off the surface using Accutase solution (Sigma-Aldrich) and subcultured in culture plates covered by fibronectin. Further, the cells were subcultured when confluence reached 70–80%. On culture day 19–22, we performed immunophenotyping and a functional analysis of the obtained culture.

Primary human coronary artery endothelial cells (HCAEC) (Cell Applications, USA) were used as control. According to the manufacturer’s information, the cells were isolated from the arteries of healthy donors with cryodestruction on the second passage (500,000 cells in MesoEndo Cell Basal Medium (Cell Applications) containing 10% fetal bovine serum and 10% dimethyl sulfoxide). The cells were unfreezed and cultured in MesoEndo Cell Growth Medium (Cell Applications) according to the manufacturer’s recommendations.

For further experiments, we used the cells of 6–8th passages. All procedures were carried out in sterile conditions, the cells being cultured in CO2 incubator at 37°C, 5% CO2.

In the experiment, ECFC and HCAEC cultures were taken off the surface and inoculated in special μ-Luer plates (IBIDI, Germany), the concentration being 1**·**106 cells/ml and precultured in an incubator for a night to form a monolayer. After that, a part of the plates were connected to a pump providing a continuous flow of culture medium, and were cultivated within 2 days at shear stress of 5 dyn/cm^2^ (dynamics). Other plates were cultured under standard conditions within the same period of time (statics). After the culturing was finished, we analysed the gene expression in the cultures under study.

### Gene expression

Total RNA was isolated from cell samples, by 4 replicates per each cell line. After culture medium withdrawal, the cells were washed with cold phosphate-buffered saline, and then lyzed by trizol (Invitrogen, USA). Total RNA was isolated using PureLink RNA Micro Scale Kit (Invitrogen) with accompanying treatment by DNAse (Sigma-Aldrich). Isolated RNA amount was assessed using NanoDrop 2000 spectrophotometer (Thermo Fisher Scientific, USA). The expression of genes *NOTCH4*, *NRP2*, *PLAT*, *PLAU*, *NOTCH1*, *FLT1*, *COL4A2*, *CD34*, *SERPINE1*, *HEY2*, *MKI67*, *KLF4*, *LYVE1*, *FLT4* was evaluated by a quantitative real-time polymerase chain reaction. The primers were synthesized by Evrogen JSC (Moscow, Russia), the sequences used are represented in the Table. Three reference genes *ACTB*, *GAPDH*, and *B2M* were used for result normalization of quantitative PCR according to accepted standards. The expression of the genes under study was calculated using ΔCt method and expressed on a logarithmic (log10) scale in the form of a fold change related to the control samples.

**Table:** Characteristics of primers used in the study

Gene	Sequence
*NOTCH4*	Forward: 5′-ACA CAG GCT CCC GTT GTG AG-3′
	Reverse: 5′-GGC ACA CTC GTT GGT CTC CA-3′
*NRP2*	Forward: 5′-CTC GGC TTT TGC AGG TGA GAA T-3′
	Reverse: 5′-TGC TCC AGT CCA CCT CGT AT-3′
*PLAT*	Forward: 5′-TGG AGC AGT CTT CGT TTC GC-3′
	Reverse: 5′-CCA TGA CTG ATG TTG CTG GTA-3′
*PLAU*	Forward: 5′-CCT GGG TCG CTC AAG GCT TA-3′
	Reverse: 5′-CAC ACC TGC CCT CCT TGG AA-3′
*NOTCH1*	Forward: 5′-GCT CAC GCT GAC GGA GTA CA-3′
	Reverse: 5′-ATG GAA GCT GGG TGG GCA GT-3′
*FLT1*	Forward: 5′-ACG GCC AGC GAG TAC AAA G-3′
	Reverse: 5′-AGT CAC GTT TGC TCT TGA GG-3′
*COL4A2*	Forward: 5′-CAG GTT TTC CGG GAC TCC GT-3′
	Reverse: 5′-AAG GGT GTT GGC CTC TCC TG-3′
*CD34*	Forward: 5′-GTC TTC CAC TCG GTG CGT CT-3′
	Reverse: 5′-TGG GGT AGC AGT ACC GTT GT-3′
*SERPINE1*	Forward: 5′-CGC CGC CTC TTC CAC AAA TC-3′
	Reverse: 5′-AGG GCA GTT CCA GGA TGT CG-3′
*HEY2*	Forward: 5′-ACC TCC GAG AGC GAC ATG GA-3′
	Reverse: 5′-CGA TCC CGA CGC CTT TTC TC-3′
*MKI67*	Forward: 5′-GAC TTG ACG AGC GGT GGT TC-3
	Reverse: 5′-GGG AAG GCC AGG TAT AAT CCG T-3′
*KLF4*	Forward: 5′-GAA AAG GAC CGC CAC CCA CA-3′
	Reverse: 5′-AGC GGG CGA ATT TCC ATC CA-3′
*LYVE1*	Forward: 5′-TGA AGG GGT AGG CAC GAT GG-3′
	Reverse: 5′-GCA TGA CAC CTG GAT GGA AAG C-3′
*FLT4*	Forward: 5′-TGT GGT CCT TTG GGG TGC TT-3′
	Reverse: 5′-CCC TCA TCC TTG TGC CGT CT-3′

### Statistical analysis

The results were processed using GraphPad Prism 6 (GraphPad Software, USA). The data were represented as a median and quartiles (Ме [25%; 75%]). The groups were compared using Mann–Whitney U test. The values with p≤0.05 were considered statistically significant. When comparing ECFC and HCAEC, significant differences in gene expression were determined by the fold change ≥2.

## Results and Discussion

**Figure 1.**
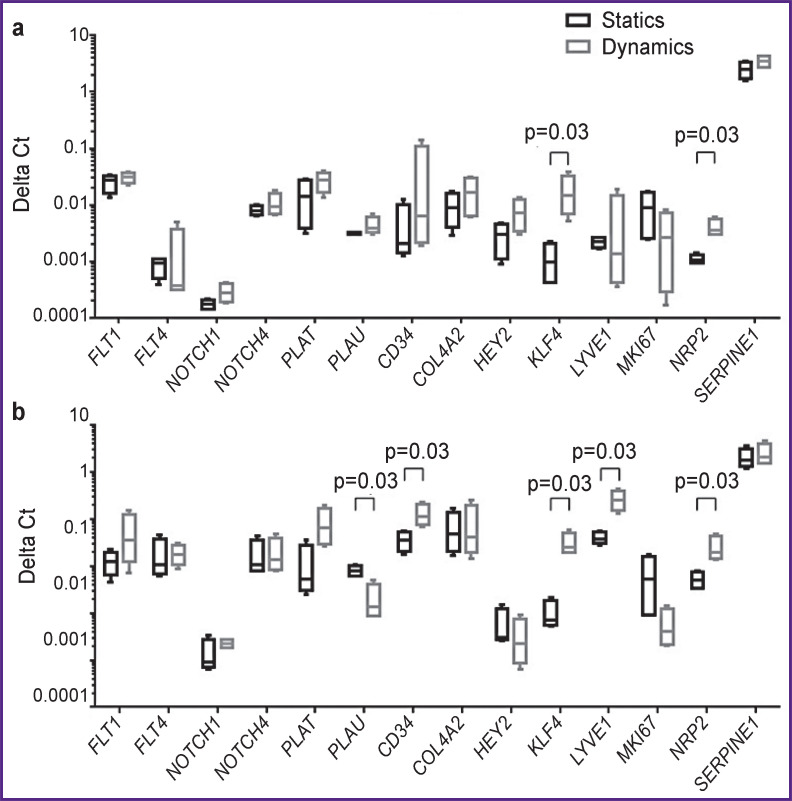
Gene expression in ECFC (а) and HCAEC (b) cultures

Mature endothelial cells (HCAEC) were found to slightly change their gene profile when exposed to a laminar flow. Thus, only the values of transcription factor *KLF4* and venous differentiation factor *NRP2* (р=0.03) (Figure 1 (a)) appeared to change significantly.

ECFC response to shear stress was more expressed. Similar to HCAEC, *KLF4* and *NRP2* increased significantly (р=0.03) (Figure 1 (b)). Moreover, there was the growth of the markers: *CD34* and lymphatic differentiation *LYVE1*, as well as the decrease of *PLAU* (urokinase plasminogen activator) expression.

Considering heterogeneity of different lines of endothelial cells, a detailed study of their secretory activity, a gene expression profile and the peculiarities of response to specific stimuli, such as shear stress, can help to determine the suitability of any endothelial cell population for cardiac and vascular tissue engineering implementation. A line of human mature arterial endothelial cells was used as control in the experiment supposing the similarity of an experimental line and the cells to indicate their possible application to solve tissue engineering problems.

Shear stress was found to cause no significant changes in HCAEC profile, except for an increased expression of a transcription factor *KLF4* related to antithrombotic and anti-inflammatory endothelial function, as well as venous endothelial marker *NRP2*. No expressed response to a laminar flow can be primarily related to low shear stress. Experimentally, low stress is frequently used when culturing *in vitro* tissue engineering constructs [13, 14]; however, *in vivo*, endothelial cells are exposed to shear stress of 5–20 dyn/cm^2^ [15]. Thus, the value we used in the experiment was at the lower borderline of a physiological standard.

ECFC were revealed to exhibit higher sensitivity to the laminar flow action. And it is particularly remarkable that along with the venous endothelial marker *NRP2* expression increase, a significant growth of *LYVE1* expression related to lymphatic endothelium differentiation was found. Moreover, shear stress caused the decrease in *PLAU* expression, which is a fibrinolytic system component but not *PLAT*, which is also involved in fibrinolysis. *PLAU* hypoexpression effect was previously shown for mature endothelial cells with normal shear stress related to static culture [16]. In our experiment, the present effect was observed only for ECFC; that can be associated with lower flow parameters and can be likely due to higher sensitivity of immature endothelial cells to altered dynamic effect.

**Figure 2.**
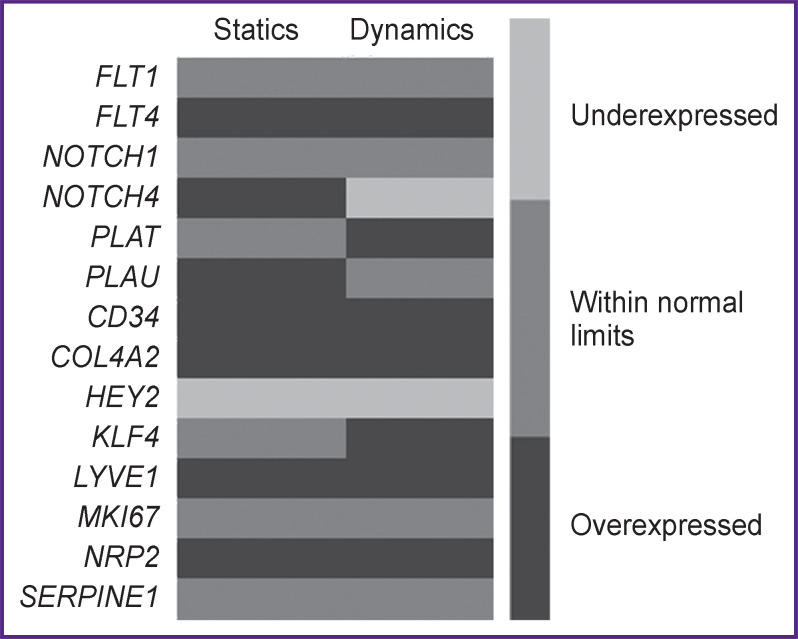
Gene expression level in ECFC culture compared to HCAEC

All genes under study were divided into three groups depending on their expression character in ECFC compared to HCAEC: the genes with overexpression, the genes with hyperexpression, the genes with no expression changes, and those with hypoexpression (Figure 2).

The comparison of ECFC and HCEAC gene expression profiles in static culture conditions confirmed an ECFC endothelial phenotype, however, it showed its transitional character, which was demonstrated in hyperexpression of *LYVE1* and *FLT4*, the markers of lymphatic endothelial differentiation, *NOTCH4* marker hyperexpression associated with the phenotype of arterial endothelial cells, and venous differentiation marker *NRP2* hyperexpression in combination with strongly marked decreased expression of *HEY2*, an arterial differentiation marker. In addition, there was *CD34* hyperexpression typical for progenitor cells. ECFC specific characteristic was an increased expression of *COL4A2* related to type IV collagen synthesis.

Under the shear stress action, the mentioned tendencies are generally preserved. And, culture in a laminar flow contributed to the increase in phenotype differences: when an expression level of panendothelial marker *FLT1* remained the same, there was *NOTCH4* hypoexpression observed. Moreover, there was *KLF4* hyperexpression found that was consistent with the data on the effect a transcriptional factor had on Notch signaling pathway [17]. Regardless of the fact that *KLF4* was associated with endothelial cell differentiation [17], and if the correlation of venous and lymphatic differentiation markers is preserved, *NOTCH4* expression changes can suggest a negative effect of a low shear stress on ECFC arterial differentiation.

ECFC are known to be able to be differentiated from a large number of different sources [5-9]. However, ECFC characteristics of different origin, as well as their response to the effect of specific stimuli are underinvestigated. Researches show that ECFC response to shear stress generally appears to be the similar to that in mature endothelial cells [18]. In our study, the profile of ECFC isolated from peripheral blood of patients with CHD turned out to be close to HCAEC; however, there were differences in the expression of certain genes associated with the differentiation of different endothelial lines, which increased under laminar flow action. Disagreement with other researches, which relate the enhancement of mature endothelium markers expression under dynamic culture in endothelial progenitor and colony-forming to shear stress effect [19], can be explained primarily by different cell origin, as well as different conditions of culture and flow parameters in different experiments.

Some researchers succeeded in endothelialization of tissue-engineered scaffold surface using low shear stress values [19, 20]. Such an approach enables to avoid (due to the reduced impact of culture conditions) the damage to a forming endothelial layer under a flow effect. However, our findings suggest the necessity of thorough selection of culture modes, at least, when using ECFC, which are going to contribute to the formation of a mature endothelial cell phenotype.

## Conclusion

The findings suggest that ECFC gene expression profile is sufficiently close to that of HCAEC, thus, ECFC isolated from peripheral blood of patients can be used to develop patient-specific tissue-engineered vascular grafts. On the other hand, further studies are necessary to determine the peculiarities of ECFC response to shear stress of different values since these data can be essential for specifying optimal conditions when forming tissue engineering constructs.
